# Adherence to the 2018 AHA cholesterol management guideline in hyperlipidemia treatment among adults in an outpatient setting

**DOI:** 10.3389/fcvm.2024.1340311

**Published:** 2024-07-10

**Authors:** Bahere Behdani, Toba Kazemi, Mahmood Zardast, Saeede Khosravi Bizhaem, Shima Jafari

**Affiliations:** ^1^ Student Research Committee, Birjand University of Medical Sciences, Birjand, Iran; ^2^ Cardiovascular Diseases Research Center, Birjand University of Medical Sciences, Birjand, Iran; ^3^ Department of Clinical Pharmacy, School of Pharmacy, Cardiovascular Diseases Research Center, Birjand University of Medical Sciences, Birjand, Iran

**Keywords:** statin, AHA Guideline, diabetes, ASCVD, hyperlipidemia

## Abstract

**Background:**

Although evidence-based guidelines and effective treatments exist for dyslipidemia, a significant disparity remains between guidelines and clinical practice. In this study, we investigated adherence to statin therapy per the 2018 ACC/AHA Guideline recommendations.

**Methods:**

This is a retrospective, descriptive-analytical study involving 1,224 individuals who presented to the laboratories located in Birjand, Eastern Iran, from June 2022 to March 2023. Analyses were conducted on 700 patients. Data collection utilized a checklist and serum value measurements of laboratory factors deemed necessary for the study.

**Results:**

Treatment was administered per the guidelines for 348 out of the 700 patients (49.7%). With 60.7%, the diabetes group exhibited the highest level of adherence to guidelines. In the atherosclerotic cardiovascular disease (ASCVD) group, 31.7% followed the recommendations. The lowest adherence rates were in groups with a 10-year ASCVD risk score of ≥20% and severe hypercholesterolemia, respectively (0% and 2.8%). In our study, atorvastatin was the most frequently prescribed statin, with the majority of patients consuming a moderate-intensity statin. None of the severely hypercholesterolemic patients achieved the LDL goal. Moreover, LDL-C goal achievement was low among the ASCVD group and those with an ASCVD risk score of ≥20%.

**Conclusion:**

Patients with hypercholesterolemia adhere inadequately to the AHA Guideline. Consequently, training courses are needed to inform medical doctors, particularly general practitioners, of the latest dyslipidemia treatment recommendations as the AHA advises.

## Introduction

Currently, atherosclerotic cardiovascular disease (ASCVD) is a significant contributor to morbidity and mortality worldwide (1). Dyslipidemia is recognized as a major contributing factor to fatal cases of cardiovascular disease (CVD), including stroke and myocardial infarction (MI), from both biological and clinical perspectives (2). Studies have shown that dyslipidemia affects nearly 30.3%, 36.7%, 47.7%, and 53.7% of the population in Southeast Asia, the Western Pacific, the Americas, and Europe, respectively (3, 4). The 2018 American College of Cardiology (ACC)/American Heart Association (AHA) Guideline recommends statins as one of the safest and most effective therapeutic agents for both ASCVD treatment and prevention (5). Various lines of evidence have firmly established that low-density lipoprotein (LDL), very low-density lipoproteins (VLDL), intermediate-density lipoproteins (IDL), and lipoprotein(a), all of which are rich in cholesterol, play a direct role in the progression of ASCVD (6). Therefore, managing dyslipidemia primarily revolves around monitoring the LDL cholesterol (LDL-C) level, which may vary based on specific diseases such as diabetes mellitus (MD) and the 10-year ASCVD risk score (7).

Statins inhibit 3-hydroxy-3-methylglutaryl coenzyme A (HMG-CoA) reductase, thereby altering cholesterol levels and reducing the chances of atherosclerosis, heart attacks, and other forms of ASCVD (8). Recent clinical studies have uncovered intriguing findings about the effectiveness of statin therapy for patients at risk of CVD. Statins have been shown to significantly reduce LDL-C levels, thereby improving the lipid profile and offering better protection against cardiovascular events for high-risk patients when compared to conventional lipid-lowering treatments (9, 10). Considering statin's properties and the existing clinical evidence, this class of anti-atherosclerosis drugs is the preferred therapeutic approach for both primary and secondary prevention of CVDs (11). , however, it is often overlooked that compliance and adherence to statin therapy are important factors.

Given the significance of compliance with therapeutic protocols for achieving optimal outcomes, several studies have been conducted worldwide to assess patients’ adherence to statin therapy. Nationwide studies conducted in the United States found that approximately 15%–40% of prescribed statins adhered to the most recent guidelines, particularly the latest ACC/AHA Guideline (12, 13). In Saudi Arabia, the adherence rate ranged from 10% to 75%, whereas in Malaysia, it ranged between 40% and 50% (14, 15). The adherence rates to guidelines differ across countries. No studies examining the adherence of Iranian patients to statin therapy were identified despite a thorough search across multiple databases and indications of adherence to statin therapy in other countries. This study aims to assess the compliance and adherence of Iranian patients in Birjand, eastern Iran, to the statin therapy as outlined in the 2018 ACC/AHA Guideline.

## Methods

### Study design

The study aimed to analyze the adherence of adults with high LDL-C levels to statin therapy according to the 2018 AHA Guideline. This cross-sectional study enrolled participants in the research from 7 June 2022 to 20 March 2023 at a private laboratory and Imam Reza Hospital laboratory department, the two facilities with the highest patient visits in the city. The research protocol was approved on 27 April 2022, under the reference number IR.BUMS.REC.1401.006. The participants provided informed consent, and data collection forms were anonymized with identification codes to ensure patient confidentiality.

### Participants and data collection

The data collected for each participant comprised demographic characteristics [e.g., gender, age, and healthcare supervisor (a specialist or general practitioner]. Moreover, data were collected on systolic and diastolic blood pressure (SBP and DBP, respectively), smoking history, and medical history, including DM, hypertension, percutaneous coronary intervention (PCI), chronic coronary syndrome (CCS), stroke, transient ischemic attack (TIA), MI, coronary artery bypass grafting (CABG), carotid artery stenosis, chronic kidney disease (CKD), anti-hypertensive medication, and statin therapy status (i.e., medication and its dose). Furthermore, specific laboratory data were collected, including total cholesterol, triglyceride (TG), LDL-C, high-density lipoprotein cholesterol (HDL-C), and fasting blood glucose (FBG) levels and the presence of CAD, CCS, and PCI.

### Settings

**Figure 1:**
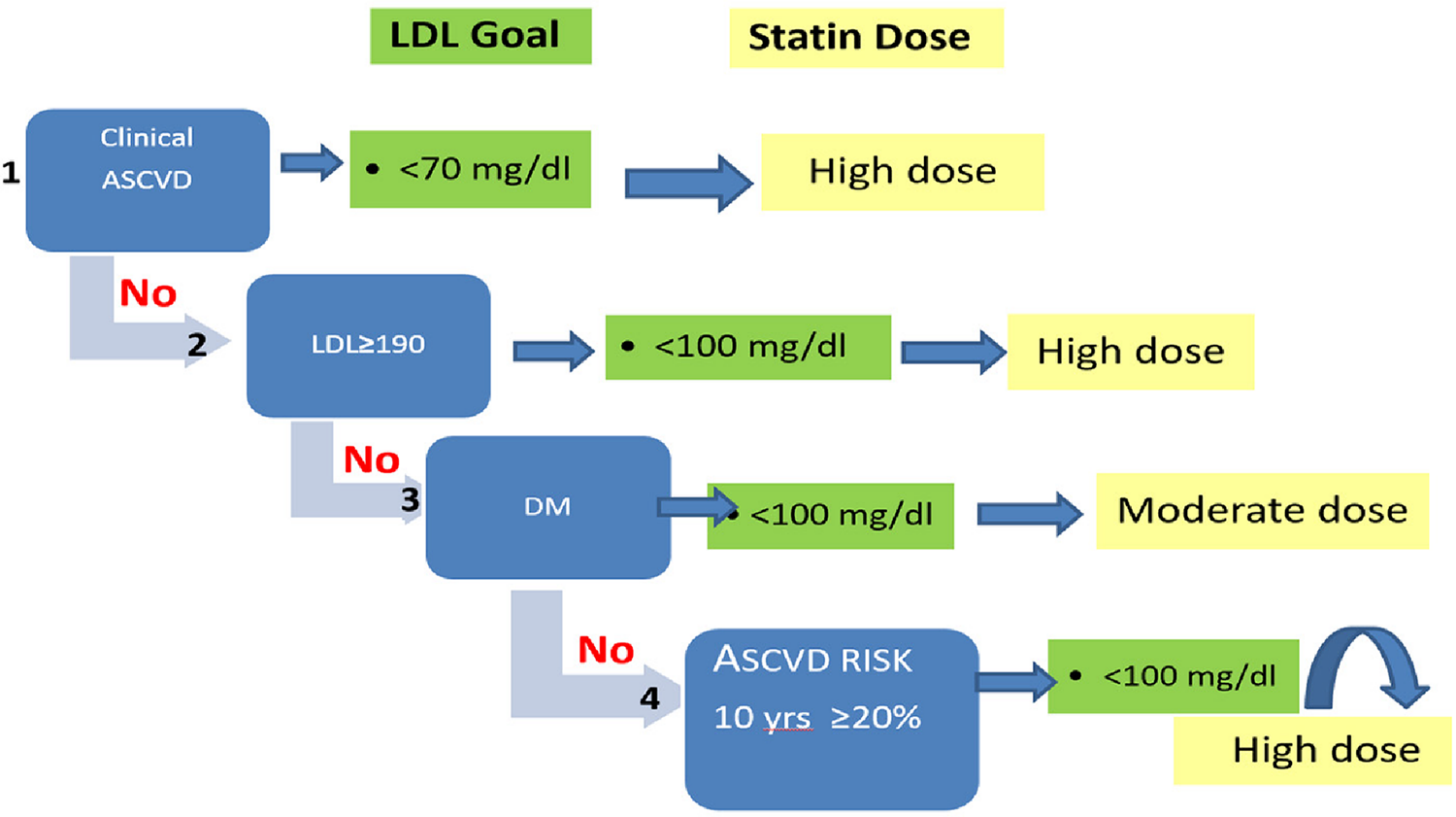
Step-by-step LDL-C approach according to the 2018 AHA Guideline.

As illustrated in Figure 1, the patients were classified into four groups in accordance with their underlying condition: (1) patients with approved ASCVD; (2) patients with LDL-C ≥ 190 mg/dl who are classified as having severe hypercholesterolemia; (3) patients with FBG ≥ 126 and/or taking glucose-lowering medications who are classified as diabetic; (4) patients with LDL-C levels between 70 and 189 mg/dl and a 10-year ASCVD risk of ≥20%.

Based on the 2018 AHA Guideline, the ASCVD risk score estimator determined the 10-year ASCVD risk by considering age, gender, SBP, DBP, cholesterol profile, smoking history, use of anti-hypertensive therapy, and family history of DM. Our study only included patients with an ASCVD risk score of ≥20% (16). The patients’ statin therapy was classified into low, moderate, and high-intensity groups according to the 2018 AHA Guideline (Table 1) (16). Table 2 lists the use of additional medications, such as ezetimibe. We assessed the degree of adherence to the guideline recommendations by considering the patient's guideline group, whether they were administered the recommended statin dosage, and whether they achieved the intended goals. We compared the patient's LDL-C level and statin dosage with the guideline's LDL-C goal and statin dosage to assess the adherence rate. An LDL-C value indicated the presence of adherence within the specified range; conversely, non-adherence was indicated by a higher statin dose or a higher LDL-C value.

**Table 1:** Statin intensity dose according to an available statin in Iran.

Intensity	High	Moderate	Low
Statins	Atorvastatin 40–80 mg Rosuvastatin 20–40 mg	Atorvastatin 10–20 mg Rosuvastatin 5–10 mg Simvastatin 20–40 mg Lovastatin 40–80 mg	Simvastatin 10 mg Lovastatin 20 mg

**Table 2:** Statin adherence according to the 2018 AHA Guideline for different drug consumers.

Drugs category	Total (*n* = 700)	Concordant (*n* = 348)	Discordant (*n* = 352)	*p*-value
Only statin	558 (79.7)	343 (61.5)	215 (38.5)	<0.001^c^
Statin + non-statin^a^	8 (1.1)	5 (62.5)	3 (37.5)	
Non-statin^a^	9 (1.3)	0	9 (100)	
None^b^	125 (17.9)	0	125 (100)	
Statin intensity				
Low	18 (2.6)	0	18 (100)	<0.001^c^
Moderate	429 (61.3)	292 (68.1)	137 (31.9)	
High	119 (17)	56 (47.1)	63 (52.9)	
None	134 (19.1)	0	134 (100)	
Goal				
Get LDL-C goal	334 (47.7)	196 (58.7)	138 (41.3)	<0.001^c^

^a^ Non-statin lipid-lowering drugs such as gemfibrozil, fenofibrate, and ezetimibe.

^b^ Not taking any lipid-lowering drugs.

^c^ Means statistically significant.

### Statistical analysis

Statistical analysis was conducted using SPSS for Windows version 22.0 (SPSS Inc., Chicago, IL, USA). Median (Q1–Q3) and frequency (%) were employed to express continuous and categorical variables, respectively. The Kolmogorov–Smirnov test was used to assess the normal/non-normal distribution of data. The chi-square test and/or Fisher's exact test compared differences in categorical variables between groups. We used the Mann–Whitney *U* test to compare unpaired samples if required. A *p*-value ≤ 0.05 was considered significant.

## Results

**Figure 2:**
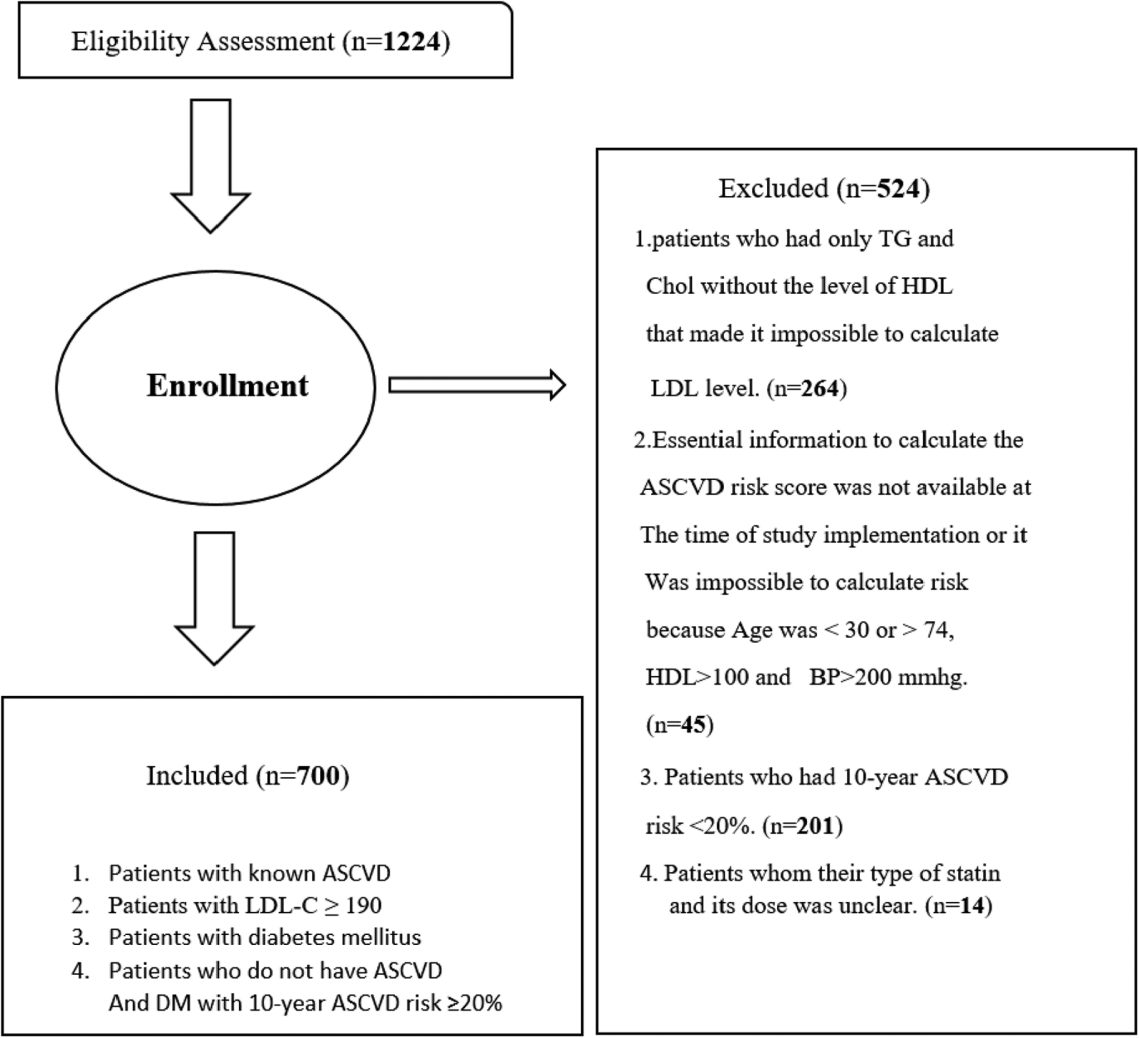
Flowchart of the derivation of study patients. *n*, number of patients; TG, triglyceride; Chol, cholesterol; HDL, high-density lipoprotein; ASCVD, atherosclerotic cardiovascular disease; LDL-C, low-density lipoprotein cholesterol; DM, diabetes mellitus; BP, blood pressure.

We enrolled 1,224 individuals referred to Imam Reza Hospital's laboratory department and a private laboratory. Among them, 264 (21.5%) individuals who had an incomplete lipid profile were excluded. Another 45 individuals had to be excluded, as it was not possible to calculate their 10-year ASCVD risk scores. Additionally, our analysis excluded 201 patients with a 10-year ASCVD risk below 20% and 14 patients with unclear prescribed statin type or dose. Thus, we analyzed 700 patients who were categorized into four groups: patients with known ASCVD, severe hypercholesterolemic patients with LDL-C ≥ 190 mg/dl, diabetic patients, and, lastly, individuals who did not have DM or known ASCVD but have an ASCVD risk score of ≥20% (Figure 2). Out of the 700 patients with a mean age of 59–60 years, the majority were female (63.7%). Most patients (*n* = 459; 65.6%) received treatment from general practitioners, while 241 (34.4%) were attended to by medical specialists. Moreover, 173 (24.7%), 35 (5%), 481 (68.7%), and 11 (1.6%) patients had known ASCVD, severe hypercholesterolemia, DM, and a 10-year ASCVD risk score of ≥20%, respectively. Nearly half of the patients (49.7%) adhered to guideline recommendations for statin therapy. A significantly greater proportion of patients in categories such as ASCVD, severe hypercholesterolemia, and a 10-year ASCVD risk score of ≥20% (68.2%, 97.1%, and 100%, respectively) were discordant with the guideline. In contrast, approximately 60% of diabetic patients were prescribed guideline-concordant statin therapy (*p* < 0.001). The variables CAD and stroke represent CAD + PCI + CABG and stroke + TIA + carotid stenosis, respectively. Table 3 compares concordance and discordance based on guidelines for various parameters, per the LDL-C management categories.

**Table 3:** The 2018 AHA Guideline concordance and discordance concerning demographics, laboratory findings, and medical history.

Variables	Total (*n* = 700)	Concordant (*n* = 348)	Discordant (*n* = 352)	*p*-value
Age (year)	60 [52–66]	59 [53–65]	60 [52–67]	0.363
Sex (male)	254 (36.3)	131 (51.6)	123 (48.4)	0.458
Sex (female)	446 (63.7)	217 (48.7)	229 (51.3)	** **
SBP (mmHg)	120 [110–130]	120 [110–130]	120 [110–130]	0.297
DBP (mmHg)	80 [70–80]	80 [70–80]	80 [70–80]	0.524
Chronic disease				
DM	626 (89.4)	335 (53.5)	291 (46.5)	<0.001^a^
HTN	359 (51.3)	175 (48.7)	184 (51.3)	0.567
CAD	235 (33.6)	82 (34.9)	153 (65.1)	<0.001^a^
Stroke	13 (1.9)	2 (15.4)	11 (84.6)	0.021^a^
Laboratory findings				
TG (mg/dl)	138 [100–187]	135 [93.25–184]	140 [107–205]	0.011^a^
Chol (mg/dl)	177 [145–213]	171 [140–200]	187 [151–227]	<0.001^a^
HDL (mg/dl)	42 [38–45]	42 [38–45]	42 [38–44]	0.743
LDL-C (mg/dl)	94 [69–127.75]	86 [65–114]	99.5 [72–139.75]	<0.001^a^
FBG (mg/dl)	119 [99–158]	121 [102–159.75]	117 [98–156.50]	0.233
LDL-C management category				
ASCVD	173 (24.7)	55 (31.8)	118 (68.2)	<0.001^a^
Severe hypercholesterolemia	35 (5)	1 (2.9)	34 (97.1)	
DM	481 (68.7)	292 (60.7)	189 (39.3)	
10-year ASCVD risk score of ≥20%	11(1.6)	0(0)	11(100)	

*n*, number of patients; SBP, systolic blood pressure; DBP, diastolic blood pressure; DM, diabetes mellitus; HTN, hypertension; CAD, coronary artery disease; TG, triglyceride; Chol, cholesterol; HDL, high-density lipoprotein; LDL-C, low-density lipoprotein; FBG, fasting blood glucose; ASCVD, atherosclerotic cardiovascular disease. Data presented as frequency (percent) and median [Q1–Q3].

^a^ Means statistically significant.

**Figure 3:**
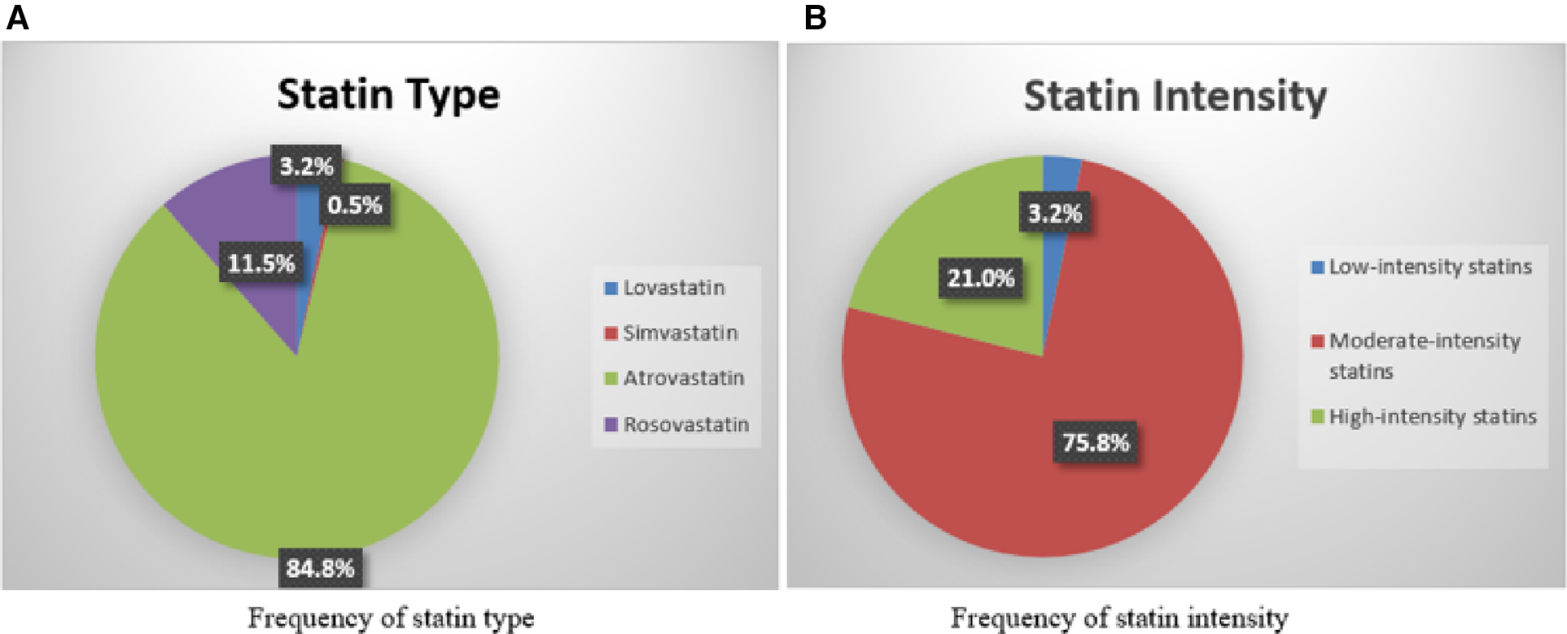
Frequency of statin type and intensity prescribed. The statin type and intensity frequency are prescribed for patients. Moderate-intensity atorvastatin was the most prescribed statin in our study. Although high-intensity statins were recommended for patients with known ASCVD, LDL-C ≥ 190.

**Figure 4:**
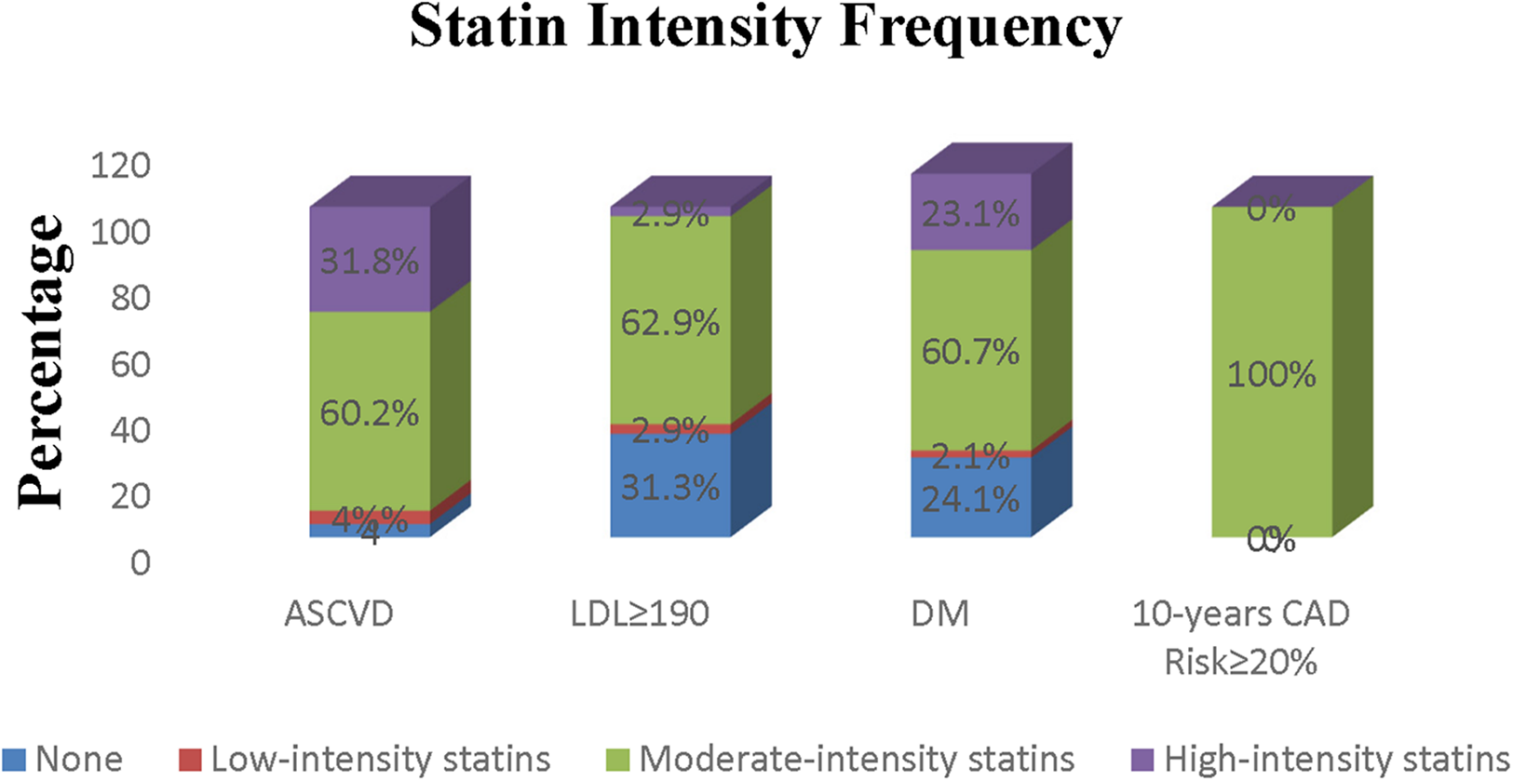
Comparison of frequency of different statin intensities prescribed in different risk groups.

Figure 3 displays the statin type and intensity prescribed for patients. Moderate-intensity atorvastatin was the most prescribed statin in our study. Although high-intensity statins were recommended for patients with known ASCVD, LDL-C ≥ 190 mg/dl, and a 10-year ASCVD risk score of ≥20%, the majority had taken moderate-intensity statin therapy (60.2%, 62.9%, and 100%, respectively). Diabetic patients were the only individuals, most of whom (60.7%) took moderate-intensity statins, as was recommended in the guideline. Figure 4 compares the statin intensity percentage of each group.

**Figure 5:**
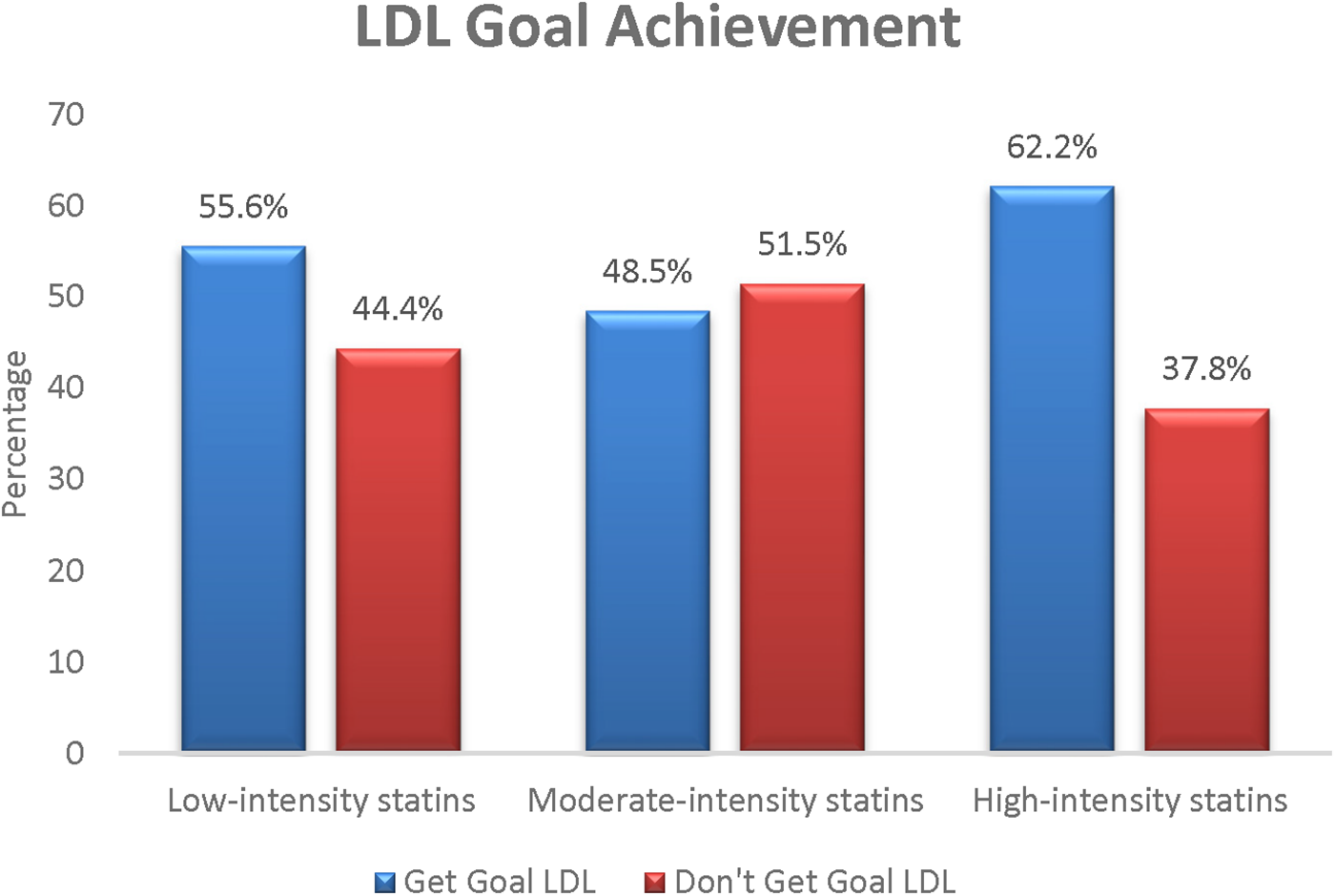
LDL-C goal achievement according to statin intensity prescribed.

Figure 5 compares the LDL-C goal achievement based on statin intensity to determine which intensity achieved the highest LDL-C goal. As expected, the highest category in this comparison was high-intensity statins, with 62.2% LDL-C goal achievement.

Based on Table 3, around 60% of patients receiving statin treatment alone or in combination with other lipid-lowering drugs (LLDs) such as gemfibrozil, fenofibrate, and ezetimibe were following the guidelines. Conversely, none of the patients using LLDs other than statins or not taking any LLDs were following the guidelines. The patients who were prescribed moderate-intensity statins were more concordant with the guidelines than the patients prescribed low-intensity statins (68.1% vs. 0%, *p* < 0.0001). Below 50% of high-intensity prescriptions (47.1%) were guideline concordant. Among the 700 patients, 334 (47.7%) achieved the guideline-recommended LDL-C goal. Out of these individuals, 196 patients (58.7%) were treated under the guidelines for LDL-C management. Among patients with known ASCVD, 34.7% achieved LDL-C < 70 mg/dl. None of the patients with severe hypercholesterolemia could achieve the LDL-C goal, and 56.5% of patients with DM alone achieved LDL-C < 100 mg/dl.

According to the 2018 AHA Guideline, LDL-C goal achievement and statin intensity recommended for each category are as follows: (1) LDL-C < 70 mg/dl and a high-intensity statin for patients with known ASCVD; (2) LDL-C < 100 mg/dl and a high-intensity statin for patients with severe hypercholesterolemia; (3) LDL-C < 100 mg/dl and moderate-intensity statin for diabetic patients; and (4) LDL-C < 100 mg/dl and high-intensity statin for patients having a 10-year ASCVD risk score of ≥20% (7). Table 4 reveals the achievement of lipid goals by statin intensity type in different risk categories.

**Table 4:** Achievement of LDL-C goals by statin intensity type in different risk categories.

	ASCVD1 *n* = 173	Severe hypercholesterolemia *N* = 35	DM *n* = 481	10-year ASCVD risk score of ≥20% *n* = 11	Total *n* = 700
LDL-C < 70	60 (34.7%)	–	119 (24.7%)	–	179 (25.6%)
None	0	–	14 (11.8)	–	14 (7.8)
Low-intensity statins	2 (3.3)	–	3 (2.5)	–	5 (2.8)
Moderate-intensity statins	34 (56.7)	–	80 (67.2)	–	114 (63.7)
High-intensity statins	24 (40)	–	22 (18.5)	–	46 (25.7)
70 < LDL-C < 100	56 (32.4%)	–	153 (31.8%)	2 (18.2%)	211 (30.1%)
None	5 (8.9)	–	28 (18.3)	0	33 (15.6)
Low-intensity statins	1 (1.8)	–	5 (3.3)	0	6 (2.8)
Moderate-intensity statins	32 (57.1)	–	92 (60.1)	2 (100)	126 (59.7)
High-intensity statins	18 (32.1)	–	28 (18.3)	0	46 (21.8)
LDL-C ≥ 100	57 (32.9%)	35 (100%)	209 (43.5%)	9 (81.8%)	310 (44.3%)
None	2 (3.5)	11 (31.4)	74 (35.4)	0	87 (28.1)
Low-intensity statins	4 (7)	1 (2.9)	2 (1)	0	7 (2.3)
Moderate-intensity statins	38 (66.7)	22 (62.9)	120 (57.4)	9 (100)	189 (61)
High-intensity statins	13 (22.8)	1 (2.9)	13 (6.2)	0	27 (8.7)

^a^ ASCVD: history of myocardial infarction, percutaneous coronary intervention, coronary artery bypass grafting, and stroke. *n*, number of patients; LDL-C, low-density lipoprotein; DM, diabetes mellitus; ASCVD, atherosclerotic cardiovascular disease.

## Discussion

The present study aimed to assess adherence to statin therapy among patients with high levels of LDL in Birjand, Eastern Iran, based on the 2018 AHA Guideline. As the central point of guideline recommendations on statin therapy, LDL-C is associated with the risk of ASCVD events linearly (17–20). In the absence of the LDL-C measurement data, the LDL-C level is calculated through the Friedewald equation. This was mentioned in a study about dyslipidemia management where LDL-C was obtained via equations rather than measured directly (17). In this study, 21.5% of individuals were excluded because of incomplete lipid profiles and inability to calculate LDL-C. This demonstrates the need for training courses for medical doctors, particularly general practitioners, who accounted for 65% of medical doctors in our study. Such training appears to be required so that medical doctors will order lipid profile tests, including TG, total cholesterol, and HDL, for calculating LDL-C levels as a cornerstone for statin therapy.

As the first line of primary and secondary prevention, statins reduce ASCVD events by lowering LDL-C (17). According to a meta-analysis of statins, MI, coronary revascularization, and all-cause mortality were reduced by 23%, 24%, and 12%, respectively, by 1 mmol/L LDL-C reduction (21). In the current study, atorvastatin was the statin most commonly used (84.8%), similar to a study conducted by Olufade et al. (12) where atorvastatin was the most prescribed statin regimen. Moreover, a previous study also reported atorvastatin as the most frequently used statin (22). One reason for this finding is that atorvastatin is covered by insurers in Iran, and it is less expensive and more accessible than other statins.

Nearly half of the patients in the present study were treated concordantly with the 2018 AHA Guideline. In another study, Ng et al. (13) reported that 65.8% of their patients were concordant with guideline recommendations. In the DA VINCI study, approximately three-quarters of 2,154 patients from six countries in Central and Eastern Europe did not achieve their LDL-C goal according to ESC 2019 guidelines (23). A study by AlAhmad et al. showed that, initially, only 60.8% of patients followed the guidelines for statin initiation and only 28.7% adhered to the recommended statin intensity. However, after interventions by clinical pharmacists, these adherence rates improved significantly (23). This shows the importance and need for clinical interventions to improve guideline adherence. Another study found that patients with an ASCVD risk score of ≥5% had 97.8% guideline concordance, whereas those with a 10-year ASCVD risk score of ≥20% had 100% discordance. Furthermore, the study found that patients with known ASCVD had the lowest rate of guideline concordance (46.9%), compared to 31.8% in our study. Moreover, consistent with our study, approximately 60% of diabetic patients in this study were concordant with guidelines (13). One explanation for this is that the frequency of DM among our patients was high (89.4%). Interestingly, most of the patients with severe hypercholesterolemia (97.1%) in our study were discordant with the guideline's recommendations, while a similar study reported a lower percentage (52%) (12).

There were no significant differences between the concordant and discordant groups in terms of gender. The findings underscore the complex interplay of demographic factors, medical history, laboratory results, drug usage, and treatment goals in determining adherence to statin therapy. Patients with chronic conditions such as DM and CAD exhibited a higher likelihood of discordance, indicating the need for tailored interventions for these subgroups. Furthermore, the discordant patients exhibited significantly higher Tg, cholesterol, and LDL-C levels.

Previous studies have provided extensive evidence of the benefits of dyslipidemia management in both primary and secondary prevention of cardiovascular events (24, 25). At the same time, studies on the compliance of patients with statin therapy show discouraging results (22). Physicians should take the initiative to improve treatment compliance. For example, frequent and regular assessment of lipid profiles could improve patients’ compliance. Previous studies have reported the ideal interval to be 1–4 times per year once the desired goal is achieved (16). Some physicians may not follow such a protocol because of their unawareness or due to financial reasons. Following up with patients is of high importance, in particular, since non-compliance is highly common among patients on statin for the secondary prevention of ASCVD (26, 27).

The findings of the current study indicate that individuals with a history of ASCVD predominantly received moderate-intensity statin therapy despite the AHA Guideline recommending high-intensity statin. This is a case of undertreatment among these patients, which suggests the non-compliance of patients with the treatment. However, this could also be explained by a lack of follow-up, given that patients have different responses to statin therapy (28, 29). Correspondingly, Arca et al. observed that out of the total statin-prescribed therapy, only 7.7% comprised high-intensity statin. Moreover, <20% of ASCVD patients (excluding those with recent ACS) were administered high-intensity statin (30). Other studies also show that high-intensity statin is prescribed to far fewer patients than recommended (13, 30). According to the results, most of the patients with severe hypercholesterolemia were undertreated by moderate-intensity statin, which opposes AHA Guideline recommendations. Interestingly, none of them could achieve the goal of LDL-C, which shows the essential need to train medical doctors, especially general practitioners, to prescribe appropriate doses of statin for these patients. Another study reported a low percentage of high-intensity statin consumption (7.7%), even in individuals with heterozygous familial hypercholesterolemia (31). Most of the diabetic patients in our study had taken moderate-intensity statin. A moderate-intensity statin is typically prescribed to patients with a 10-year ASCVD risk score of ≥20%, regardless of their achieved LDL-C level. These findings indicate that the majority of non-diabetic patients in this study were undertreated, with medical doctors failing to comply with and adhere to AHA Guideline recommendations as the primary reason for failure to meet the goal.

Previous studies have reported various benefits for compliance with statins. Bitton et al. (32) reported that compliance with statins improves health and decreases costs. Another study found that the higher the statin compliance, the lower the death rate from all causes among ASCVD patients (33).

Overall, the results of our study revealed that more than half of the study subjects did not reach the desired goal. Moreover, only 34.7% of patients with known ASCVD achieved LDL-C < 70 mg/dl, while 32.4% reached 70 < LDL-C < 100 mg/dl, and even 32.9% had LDL-C ≥ 100 mg/d, with moderate-intensity statin being the predominant intensity. It is crucial to optimize statin strategies in these patients to achieve the maximum reduction in cardiovascular outcomes (30). Approximately 17.9% of ASCVD patients failed to achieve LDL-C < 70 mg/dl, although they had taken high-intensity statin. This may happen because of poor adherence to a statin regimen by patients. Similarly, other studies report patients who, despite taking high-intensity statin, do not achieve LDL-C goals (31, 34). None of the patients with severe hypercholesterolemia reached their goals. The study by Arca et al. examined heterozygous familial hypercholesterolemia, and likewise, most subjects did not reach the desired LDL-C goal (30).

In addition, 56.5% of diabetic patients achieved LDL-C < 100 mg/dl. Arca et al. (31) reported almost the same percentage (56.9%) of LDL-C goal achievement in this group. Interestingly, 63.6% of patients failed to achieve LDL-C < 100 mg/dl while taking moderate to high-dose statin. One reason for this result is poor compliance among these patients. Most of the patients with a 10-year ASCVD risk score of ≥20% did not achieve the LDL-C goal. The low rate of goal achievement among all groups in this study highlights the importance of a follow-up plan since an insufficient response to statin therapy is associated with a higher risk of coronary heart disease (35).

Based on the baseline data of the SANTORINI study, a considerable number of patients remain at high cardiovascular risk because of the insufficient implementation of the 2019 ESC/EAS guidelines. Despite being widely used across Europe to categorize patients according to their cardiovascular risk level, the guidelines are not fully applied. Insufficient risk assessment and inadequate combination treatments may contribute to this issue (36).

### Limitations

Due to its cross-sectional design, the study could not establish causal relationships or track changes over time, potentially limiting the ability to infer the direction of relationships between variables. In contrast, assessing every risk enhancer, such as the patients’ primary LDL-C levels, was not feasible as an inadequate amount of information was available. We obtained the LDL-C level only subsequent to the start of statin therapy when the patients had visited laboratories to perform tests. Furthermore, the accuracy of historical medical records and patient self-reports can differ, impacting the reliability of the findings. Since the patients were not followed up, it was impossible to assess statin intolerance. As per the AHA Guideline, additional medications such as ezetimibe and PCSK9 inhibitors should be considered if the patient fails to reach the LDL target with a high-dose statin. The patients did not receive PCSK9 inhibitor medications because their physicians appeared to be unfamiliar with the AHA Guideline, and the medications were costly. Therefore, it is crucial to offer educational programs customized for physicians.

## Conclusion

The findings of this study provided valuable insights into the adherence to the 2018 AHA Guideline among patients with high LDL-C levels. The study demonstrated a low adherence rate of approximately 50% in the study population. Most of the patients in this study were undertreated, using moderate-intensity statin. High-intensity statin consumption was low, especially among patients with severe hypercholesterolemia. Interestingly, none of the patients with severe hypercholesterolemia achieved the LDL-C goal. Moreover, the attainment of the LDL-C goals was low among the ASCVD population and patients with an ASCVD risk score of ≥20%. Therefore, it is evident that training courses are essential for medical doctors, particularly general practitioners, to stay informed about the latest recommendations for dyslipidemia treatment.

## Data Availability

The raw data supporting the conclusions of this article will be made available by the authors, without undue reservation.

## References

[1] Kim H Kim S Han S Rane PP Fox KM Qian Y Prevalence and incidence of atherosclerotic cardiovascular disease and its risk factors in Korea: a nationwide population-based study. BMC Public Health. (2019) 19(1):1112. 10.1186/s12889-019-7439-0 31412823 PMC6694551

[2] Hedayatnia M Asadi Z Zare-Feyzabadi R Yaghooti-Khorasani M Ghazizadeh H Ghaffarian-Zirak R Dyslipidemia and cardiovascular disease risk among the MASHAD study population. Lipids Health Dis. (2020) 19(1):42. 10.1186/s12944-020-01204-y 32178672 PMC7075010

[3] Smith DG. Epidemiology of dyslipidemia and economic burden on the healthcare system. Am J Manag Care. (2007) 13(Suppl 3):S68–71. . 17596114

[4] Lin C-F Chang Y-H Chien S-C Lin Y-H Yeh H-Y. Epidemiology of dyslipidemia in the Asia Pacific Region. Int J Gerontol. (2018) 12(1):2–6. 10.1016/j.ijge.2018.02.010

[5] Underberg J Toth PP Rodriguez FJPM. LDL-C target attainment in secondary prevention of ASCVD in the United States: barriers, consequences of nonachievement, and strategies to reach goals. Postgrad Med. (2022) 134(8):752–62. 10.1080/00325481.2022.2117498 36004573

[6] Ference BA Ginsberg HN Graham I Ray KK Packard CJ Bruckert E Low-density lipoproteins cause atherosclerotic cardiovascular disease. 1. Evidence from genetic, epidemiologic, and clinical studies. A consensus statement from the European Atherosclerosis Society Consensus Panel. Eur Heart J. (2017) 38(32):2459–72. 10.1093/eurheartj/ehx144 28444290 PMC5837225

[7] Cífková R Krajčoviechová A. Dyslipidemia and cardiovascular disease in women. Curr Cardiol Rep. (2015) 17:1–10. 10.1007/s11886-015-0609-5 26026998

[8] Tsimikas S Gordts PL Nora C Yeang C Witztum J. Statin therapy increases lipoprotein (a) levels. Eur Heart J. (2020) 41(24):2275–84. 10.1093/eurheartj/ehz310 31111151

[9] Li M Wang X Li X Chen H Hu Y Zhang X Statins for the primary prevention of coronary heart disease. Biomed Res Int. (2019) 2019. 10.1155/2019/4870350 30834266 PMC6374814

[10] Aghasizadeh M Bizhaem SK Baniasadi M Khazdair MR Kazemi T. Evaluation of LDL goal achievement in statin consumption, South East of Iran. Sci Rep. (2021) 11(1):10786. 10.1038/s41598-021-90228-0 34031484 PMC8144405

[11] Pang J Chan DC Watts GF. The knowns and unknowns of contemporary statin therapy for familial hypercholesterolemia. Curr Atheroscler Rep. (2020) 22(11):64. 10.1007/s11883-020-00884-2 32870376 PMC7459268

[12] Olufade T Zhou S Anzalone D Kern DM Tunceli O Cziraky MJ Initiation patterns of statins in the 2 years after release of the 2013 American College of Cardiology/American Heart Association (ACC/AHA) cholesterol management guideline in a large US health plan. J Am Heart Assoc. (2017) 6(5):e005205. 10.1161/JAHA.116.005205 28473405 PMC5524081

[13] Ng C Chung P Toderika Y Cheng-Lai A-s. Evaluation of adherence to current guidelines for treatment of hyperlipidemia in adults in an outpatient setting. Am J Health Syst Pharm. (2016) 73(23_Supplement_6):S133–S40. 10.2146/ajhp160050 27864236

[14] Aburuz S Al-Bekairy A Alqahtani A-A Harbi K Al Nuhait M Khoja A Comparison of the application of treatment panel III and American College of Cardiology/American Heart Association guidelines for blood cholesterol treatment in Saudi Arabia. J Saudi Heart Assoc. (2018) 30(4):349–55. 10.1016/j.jsha.2018.08.003 30228788 PMC6140824

[15] Zheng LJ Abdullah AS Idris NA Yuna SNM Ab Aziz MIJEB. Evaluation of utilisation and concordance to guideline-recommended statin therapy at Medical Outpatient Clinic, Hospital Kuala Krai. Pharm Res Rep. (2018) 1:42–52.

[16] Grundy SM Stone NJ Bailey AL Beam C Birtcher KK Blumenthal RS 2018 AHA/ACC/AACVPR/AAPA/ABC/ACPM/ADA/AGS/APhA/ASPC/NLA/PCNA guideline on the management of blood cholesterol: executive summary: a report of the American College of Cardiology/American Heart Association task force on clinical practice guidelines. J Am Coll Cardiol. (2019) l(24):3168–209. 10.1016/j.jacc.2018.11.002 30423391

[17] Ferraro RA Leucker T Martin SS Banach M Jones SR Toth PP. Contemporary management of dyslipidemia. Drugs. (2022) 82(5):559–76. 10.1007/s40265-022-01691-6 35303294 PMC8931779

[18] Wolska A Remaley AT. Measuring LDL-cholesterol: what is the best way to do it? Curr Opin Cardiol. (2020) 35(4):405–11. 10.1097/HCO.0000000000000740 32412961 PMC7360339

[19] Michos ED McEvoy JW Blumenthal RS. Lipid management for the prevention of atherosclerotic cardiovascular disease. N Engl J Med. (2019) 381(16):1557–67. 10.1056/NEJMra1806939 31618541

[20] Descamps OS Verhaegen A Demeure F Langlois M Rietzschel E Mertens A Evolving concepts on the management of dyslipidaemia. Acta Clin Belg. (2020) 75(1):80–90. 10.1080/17843286.2019.1702823 31846601

[21] Baigent C Keech A Kearney P Blackwell L Buck G Pollicino C Cholesterol treatment trialists’ (CTT) collaborators. Efficacy and safety of cholesterol-lowering treatment: prospective meta-analysis of data from 90,056 participants in 14 randomised trials of statins. Lancet 366(9493):1267–1278. Erratum in: lancet 2005 Oct 15–21;366(9494):1358. Lancet. (2005) 366:1267–78. 10.1016/S0140-6736(05)67394-1 16214597

[22] Gharaibeh L Al Zoubi S Sartawi H Ayyad D Al-Hawamdeh M Alrashdan R. The appropriateness of the use of statins for the secondary and primary prevention of atherosclerotic cardiovascular disease: a cross-sectional study from Jordan. Eur Rev Med Pharmacol Sci. (2023) 27(12):5480–92. 10.26355/eurrev_202306_32785 37401284

[23] Vrablik M Seifert B Parkhomenko A Banach M Jóźwiak JJ Kiss RG Lipid-lowering therapy use in primary and secondary care in Central and Eastern Europe: DA VINCI observational study. Atherosclerosis. (2021) 334:66–75. 10.1016/j.atherosclerosis.2021.08.035 34482090

[24] Taylor F Ward K Moore TH Burke M Davey Smith G Casas JP Statins for the primary prevention of cardiovascular disease. Cochrane Database Syst Rev. (2011) 1:Cd004816. 10.1002/14651858.CD004816.pub5 PMC4164175 21249663

[25] Thavendiranathan P Bagai A Brookhart MA Choudhry NK. Primary prevention of cardiovascular diseases with statin therapy: a meta-analysis of randomized controlled trials. Arch Intern Med. (2006) 166(21):2307–13. 10.1001/archinte.166.21.2307 17130382

[26] Hirsh BJ Smilowitz NR Rosenson RS Fuster V Sperling LS. Utilization of and adherence to guideline-recommended lipid-lowering therapy after acute coronary syndrome: opportunities for improvement. J Am Coll Cardiol. (2015) 66(2):184–92. 10.1016/j.jacc.2015.05.030 26160634

[27] Lee M Saver JL Wu YL Tang SC Lee JD Rao NM Utilization of statins beyond the initial period after stroke and 1-year risk of recurrent stroke. J Am Heart Assoc. (2017) 6(8):e005658. 10.1161/JAHA.117.005658 28768645 PMC5586426

[28] Simon JA Lin F Hulley SB Blanche PJ Waters D Shiboski S Phenotypic predictors of response to simvastatin therapy among African-Americans and Caucasians: the cholesterol and pharmacogenetics (CAP) study. Am J Cardiol. (2006) 97(6):843–50. 10.1016/j.amjcard.2005.09.134 16516587

[29] Kataoka Y St John J Wolski K Uno K Puri R Tuzcu EM Atheroma progression in hyporesponders to statin therapy. Arterioscler Thromb Vasc Biol. (2015) 35(4):990–5. 10.1161/ATVBAHA.114.304477 25722430

[30] AlAhmad MM ZainAlAbdin S AlAhmad K AlAhmad I AbuRuz S. Value of the clinical pharmacist interventions in the application of the American College of Cardiology (ACC/AHA) 2018 guideline for cholesterol management. PLoS One. (2023) 18(3):e0283369. 10.1371/journal.pone.0283369 36972252 PMC10042341

[31] Arca M Ansell D Averna M Fanelli F Gorcyca K Iorga Ş Statin utilization and lipid goal attainment in high or very-high cardiovascular risk patients: insights from Italian general practice. Atherosclerosis. (2018) 271:120–7. 10.1016/j.atherosclerosis.2018.02.024 29499359

[32] Bitton A Choudhry NK Matlin OS Swanton K Shrank WH. The impact of medication adherence on coronary artery disease costs and outcomes: a systematic review. Am J Med. (2013) 126(4):357.e7–.e27. 10.1016/j.amjmed.2012.09.004 23507208

[33] Rodriguez F Maron DJ Knowles JW Virani SS Lin S Heidenreich PA. Association of statin adherence with mortality in patients with atherosclerotic cardiovascular disease. JAMA Cardiology. (2019) 4(3):206–13. 10.1001/jamacardio.2018.4936 30758506 PMC6439552

[34] Perez de Isla L Alonso R Watts GF Mata N Saltijeral Cerezo A Muñiz O Attainment of LDL-cholesterol treatment goals in patients with familial hypercholesterolemia: 5-year SAFEHEART registry follow-up. J Am Coll Cardiol. (2016) 67(11):1278–85. 10.1016/j.jacc.2016.01.008 26988947

[35] Akyea RK Kai J Qureshi N Iyen B Weng SF. Sub-optimal cholesterol response to initiation of statins and future risk of cardiovascular disease. Heart. (2019) 105(13):975–81. 10.1136/heartjnl-2018-314253 30988003 PMC6582718

[36] Ray KK Haq I Bilitou A Manu MC Burden A Aguiar C Treatment gaps in the implementation of LDL cholesterol control among high-and very high-risk patients in Europe between 2020 and 2021: the multinational observational SANTORINI study. Lancet Reg Health Eur. (2023) 29:100624. 10.1016/j.lanepe.2023.100624 37090089 PMC10119631

